# Reducing scan time in ^177^Lu planar scintigraphy using convolutional neural network: A Monte Carlo simulation study

**DOI:** 10.1002/acm2.14056

**Published:** 2023-06-01

**Authors:** Ching‐Ching Yang, Kuan‐Yin Ko, Pei‐Yao Lin

**Affiliations:** ^1^ Department of Medical Imaging and Radiological Sciences Kaohsiung Medical University Kaohsiung Taiwan; ^2^ Department of Medical Research Kaohsiung Medical University Chung‐Ho Memorial Hospital Kaohsiung Taiwan; ^3^ Department of Nuclear Medicine National Taiwan University Cancer Center Taipei Taiwan; ^4^ Graduate Institute of Clinical Medicine College of Medicine National Taiwan University Taipei Taiwan

**Keywords:** ^177^Lu planar scintigraphy, convolutional neural network, Monte Carlo simulation, personalized dosimetry, scan time reduction

## Abstract

**Purpose:**

The aim of this study was to reduce scan time in ^177^Lu planar scintigraphy through the use of convolutional neural network (CNN) to facilitate personalized dosimetry for ^177^Lu‐based peptide receptor radionuclide therapy.

**Methods:**

The CNN model used in this work was based on DenseNet, and the training and testing datasets were generated from Monte Carlo simulation. The CNN input images (IMG_input_) consisted of ^177^Lu planar scintigraphy that contained 10–90% of the total photon counts, while the corresponding full‐count images (IMG^100%^) were used as the CNN label images. Two‐sample *t*‐test was conducted to compare the difference in pixel intensities within region of interest between IMG^100%^ and CNN output images (IMG_output_).

**Results:**

No difference was found in IMG_output_ for rods with diameters ranging from 13 to 33 mm in the Derenzo phantom with a target‐to‐background ratio of 20:1, while statistically significant differences were found in IMG_output_ for the 10‐mm diameter rods when IMG_input_ containing 10% to 60% of the total photon counts were denoised. Statistically significant differences were found in IMG_output_ for both right and left kidneys in the NCAT phantom when IMG_input_ containing 10% of the total photon counts were denoised. No statistically significant differences were found in IMG_output_ for any other source organs in the NCAT phantom.

**Conclusion:**

Our results showed that the proposed method can reduce scan time by up to 70% for objects larger than 13 mm, making it a useful tool for personalized dosimetry in ^177^Lu‐based peptide receptor radionuclide therapy in clinical practice.

## 1 INTRODUCTION

^177^Lu‐labeled DOTATATE targeted radionuclide therapy has shown promising results in treating neuroendocrine tumors (NETs).^1^, ^2^, ^3^ The beta transition of ^177^Lu to the ground state of ^177^Hf has a maximum beta energy of 498 keV, which results in a tissue penetration of less than 2 mm. Thus, its proper emission type makes ^177^Lu a suitable radiopharmaceutical for killing targeted cancer cells while sparing surrounding normal tissues. In addition to its short‐range beta particles, ^177^Lu also emits 208 keV gamma radiation through an isomeric transition, making it possible to gather information on tumor localization and personalized dosimetry.^4^, ^5^ In the Medical Internal Radiation Dose (MIRD) schema, the absorbed dose *D* to target organ *j* from source organ *k* is given by:

(1) $$D\left(j\right)=\sum_{k}\tilde{A}\left(k\right)\times S\left(j\leftarrow k\right)\;$$

$$\mathrm{Gy}\;\mathrm{Bq}\cdot \sec\;\frac{\mathrm{Gy}}{\mathrm{Bq}\cdot \sec}$$

$\tilde{A}\left(k\right)$ is the cumulated activity in source organ *k*, which can be derived from ^177^Lu planar scintigraphy. S(j←k) is the absorbed dose in target organ *j* per unit cumulated activity in source organ *k*, which can be found in MIRD pamphlets.^6^, ^7^, ^8^, ^9^ By theory, the cumulated activity is obtained by integrating time‐activity curve from time 0 to infinity. In our routine clinical practice, time‐activity curve is obtained using a hybrid scenario that combines a series of whole body scintigraphies acquired at different time points after the administration of radioactive drug with a single SPECT/CT.^10^ The SPECT/CT data are used to define volumes‐of‐interest, which are projected onto whole body planar scintigraphies to measure activity retained in source organs over time. The scan speed for planar scintigraphy is 10 cm/min, so a whole procedure lasts about 20 min for each planar scan. Reducing scan time can increase patient comfort and improve the registration accuracy of whole body planar scintigraphies taken at different time points, which are crucial for precise dose calculation. However, reducing scan time would increase statistical variation and may cause inaccuracy in activity measurements. Deep learning has been used in various imaging applications, and one of them is image denoising.^11^, ^12^ A more complex model can learn more intricate features from images, but the vanishing gradient problem is a common challenge in training deep neural networks. The dense convolutional network (DenseNet) model proposed by Tong et al. employed dense skip connections to alleviate the vanishing‐gradient problem and enhance the feature propagation in deep networks.^13^ This study investigated the potential of using DenseNet to reduce scan time in ^177^Lu planar scintigraphy, in order to facilitate personalized dosimetry for ^177^Lu‐based peptide receptor radionuclide therapy. Due to the rarity of NETs, the dataset of ^177^Lu planar scintigraphy scans from real patients at our institution may not be large enough to build a convolutional neural network (CNN)‐based denoising method. Hence, Monte Carlo simulation was used to generate training and testing datasets in this work.

## 2 METHODS

### 2.1 CNN model

**FIGURE 1:**
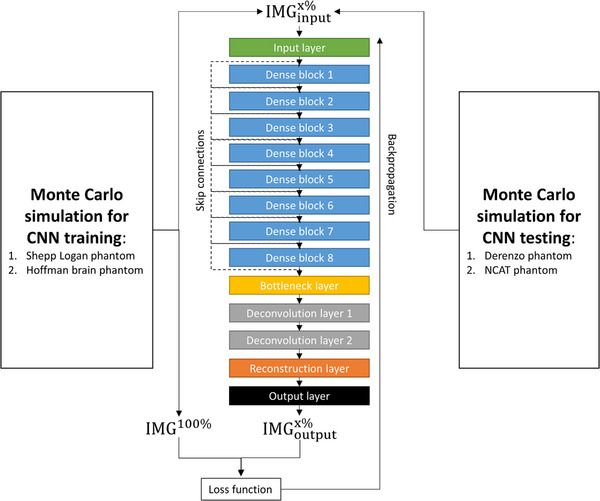
Flowchart of DenseNet training and testing for reducing scan time in ^177^Lu planar scintigraphy. ${\mathrm{IMG}}_{\mathrm{input}}^{x\%}$: CNN input images that contained x% of the total photon counts; ${\mathrm{IMG}}_{\mathrm{output}}^{x\%}$: CNN output images generated by denoising ${\mathrm{IMG}}_{\mathrm{input}}^{x\%}$; IMG^100%^: full‐count images.

The DenseNet model adopted in this study contains one convolution layer to learn low‐level features, eight DenseNet blocks to extract high‐level features, one bottleneck layer to maintain compactness and reduce computation cost, two deconvolution layers to learn upscaling filters, and one reconstruction layer to generate the output images.^13^ There are eight convolution layers in each DenseNet block, and all levels of features are combined via skip connections as input for reconstructing the output images. The CNN input images (IMG_input_) consisted of ^177^Lu planar scintigraphy containing 10% to 90% of the total photon counts, while the corresponding full‐count images (IMG^100%^) were used as the CNN label images. The root mean square error (RMSE), also known as the Euclidean distance, was the loss function used in our deep learning model to minimize the difference IMG^100%^ and IMG_input_ containing 10%−90% of the total photon counts. Using RMSE as the loss function favors a high peak signal‐to‐noise ratio (PSNR). The DenseNet model was trained from scratch. The filter weights of each layer were initialized by using the MSRA (Microsoft Research Asia) filler.^14^ All biases were initialized with zero. The models were trained by using Adam (adaptive moment estimation) optimizer with mini‐batch size of 32, learning rate of 0.0001, momentum of 0.9, and weight decay of 0.0001.^15^ Figure 1 demonstrates the flowchart of DenseNet training and testing for reducing scan time in ^177^Lu planar scintigraphy. IMG_input_ and IMG^100%^ were prepared as 25 × 25‐pixel sub‐images randomly cropped from the simulated ^177^Lu planar scintigraphies with a stride of 25 pixels to prevent overlapped image segmentation, while the matrix size of Lu‐177 planar scintigraphy was 256 × 256. There were 300 simulations performed to generate training datasets. The sub‐images containing no foreground were discarded, so the training datasets provided roughly 22 547 sub‐images. The CNN model was built, trained and tested by using Caffe (Convolutional Architecture for Fast Feature Embedding) CNN platform (version 1.0.0‐rc5 with CUDA 8.0.61) on an Ubuntu server (version 16.04.4 LTS) with two RTX 2080 (NVIDIA) graphics cards.^16^

### 2.2 Monte Carlo simulation of ^177^Lu planar scintigraphy

A Discovery NM/CT 870 DR (GE Healthcare, Milwaukee, Wisconsin, USA) was modeled by using GATE (GEANT4 Application for Tomographic Emission) 6.0.0.^17^ The imaging system modeled in this work was a dual‐head gamma camera with 3/8 inch NaI(Tl) crystal. The useful field of view (UFOV) of the system was 540 mm (transaxial) × 400 mm (axial). ^177^Lu planar scintigraphy was simulated by using 20% energy window at 208 keV and medium energy general purpose (MEGP) collimator with hexagonal holes. The MEGP collimator was built with a hole diameter of 3.0 mm, a septal thickness of 1.05 mm, and a hole length of 58 mm. With the MEGP collimator, the system sensitivity was 65 cps/MBq, while the system resolution was 9.4 mm in full width at half maximum (FWHM). A scan time of 5 min was assumed in ^177^Lu simulations. Once simulation was completed, the physics list of detected photons was stored in an ASCII (American Standard Code for Information Interchange) file, which recorded time, energy, detector location, and information about the interaction process. The detected photons in list mode were then framed into anterior and posterior projections with pixel size of 2.2 mm × 2.2 mm.

### 2.3 Phantom data for CNN training

**FIGURE 2:**
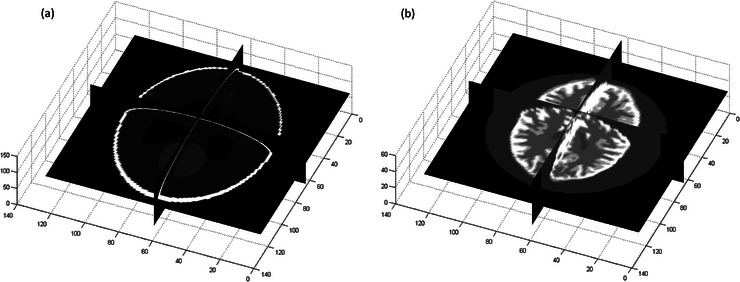
Phantoms for CNN training: (a) the Shepp Logan phantom and (b) the Hoffman brain phantom.

Figure 2a shows the Shepp Logan phantom, and Figure 2b shows the Hoffman brain phantom. Both 3D phantoms were first interpolated into a matrix size of 256 × 256 × 150. For each single axial slice, 30 duplicates were created to form an image matrix of 256 × 256 × 30. This process was repeated for all axial slices of both interpolated 3D phantoms, resulting in a total of 300 image sets. These digital phantoms were used to generate simulation data for training the CNN. The ^177^Lu planar scintigraphy for these digital phantoms was generated with a uniform water‐equivalent attenuation map in GATE Monte Carlo simulation. With regard to activity map, the activity of 150 digital phantoms created based on the Shepp Logan phantom ranged from 2.88 to 20.6 MBq, while the activity of 150 digital phantoms created based on the Hoffman brain phantom ranged from 1.36 to 31 MBq.

### 2.4 Phantom data for CNN testing

**FIGURE 3:**
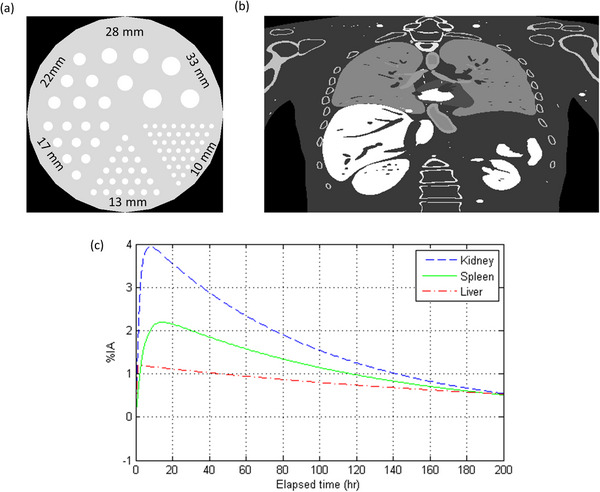
Phantoms for CNN testing: (a) the Derenzo phantom, (b) the NCAT phantom, and (c) time‐activity curves for the NCAT phantom.

The Derenzo phantom shown in Figure 3a had 340 mm diameter and was 78 mm height, so the volume of the Derenzo phantom was approximately 7000 cm^3^. The Derenzo phantom contained six rods with diameter of 10, 13, 17, 22, 28, and 33 mm, and the total volume of these rods was around 180 cm^3^. The ^177^Lu planar scintigraphy of the Derenzo phantom was generated with a uniform water‐equivalent attenuation map in GATE Monte Carlo simulation. With regard to activity map, the activity concentration of the rods was 10 MBq/kg, so the activity of Derenzo phantom with a target‐to‐background ratio (TBR) of 5:1 was 15.44 MBq, which was 5.21 MBq for the Derenzo phantom with a TBR of 20:1. Figure 3b shows the NCAT phantom, which provided a realistic model of the human anatomy.^18^ The ^177^Lu planar scintigraphy of the NCAT phantom was generated with tissue compositions recommended by ICRU (International Commission on Radiation Units and Measurements) Report 44 in GATE Monte Carlo simulation.^19^ With regard to activity map, the time‐activity curves expressed in percent injected activity (%IA) for kidney, spleen and liver measured from real patient scans are shown in Figure 3c.

### 2.5 Quantitative analysis

The difference between IMG^100%^ and CNN output images (IMG_output_) was quantified by using RMSE, PSNR, and structural similarity index measure (SSIM).

(2) $$\mathrm{RMSE}\;=\sqrt{\frac{\sum_{i=1}^{V}{\left({\mathrm{IMG}}^{100\%}-{\mathrm{IMG}}_{\mathrm{output}}\right)}^{2}}{V}}\;$$

where V was the number of voxels within the whole image.

(3) $$\mathrm{PSNR}\;=\;20\log_{10}\frac{I_{\max}}{\mathrm{RMSE}}$$

where I_max_ was the maximum intensity value of the image.

(4) $$\mathrm{SSIM}\;=\frac{\left(2\mu_{{x\mu }_{y}}+C_{1}\right)\left(2\sigma_{\mathrm{xy}}+C_{2}\right)}{\left(\mu_{x}^{2}+\mu_{y}^{2}+C_{1}\right)\left(\sigma_{x}^{2}+\sigma_{y}^{2}+C_{2}\right)}\;$$

μ_x_ and σ_x_ were the mean and standard deviation (SD) of IMG^100%^. μ_y_ and σ_y_ were the mean and SD of IMG_output_. σ_xy_ was the covariance of IMG^100%^ and IMG_output_. C_1_ and C_2_ were small constants to stabilize the division with weak denominator. RMSE, PSNR and SSIM provided a measure of image quality over the whole image.

To evaluate the quality and reliability of ^177^Lu planar scintigraphy within local sub‐regions, region of interest (ROI) analysis was performed to evaluate IMG^100%^ (low noise), IMG_input_ (high noise), and IMG_output_ (denoising). ROIs were drawn on the rods of Derenzo phantom by using the Otsu thresholding method and were drawn manually on the source organs in NCAT phantom to calculate the mean and SD within ROIs.^20^ In addition, two‐sample *t*‐test was conducted to compare the difference in pixel intensities within ROIs between IMG^100%^ and the others. A *P* value less than 0.01 was considered to be statistically significant. The data analysis processes mentioned above were conducted by using MATLAB 7.1 (The Mathworks, Natick, Massachusetts, USA).

## 3 RESULTS

### 3.1 CNN training

**FIGURE 4:**
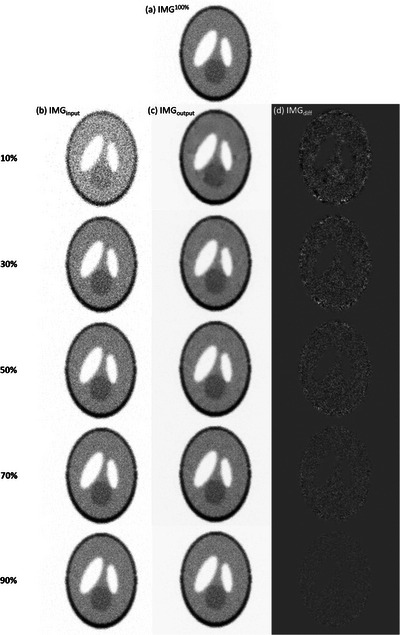
(a) IMG^100%^ for the Shepp Logan phantom, (b) IMG_input_ containing 10%, 30%, 50%, 70%, and 90% of the total photon counts (from top to bottom) and the corresponding (c) IMG_output_ and (d) IMG_diff_.

**FIGURE 5:**
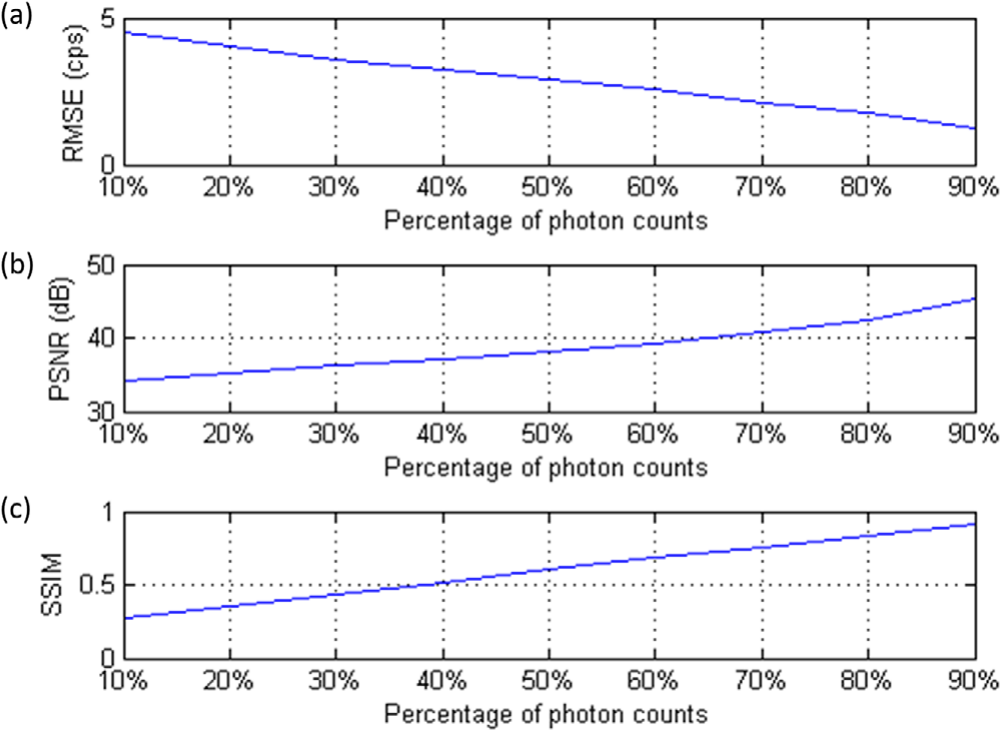
(a) RMSE, (b) PSNR, (c) SSIM between IMG^100%^ and IMG_output_ generated by denoising IMG_input_, which contained 10% to 90% of the total photon counts for the Shepp Logan phantom.

**FIGURE 6:**
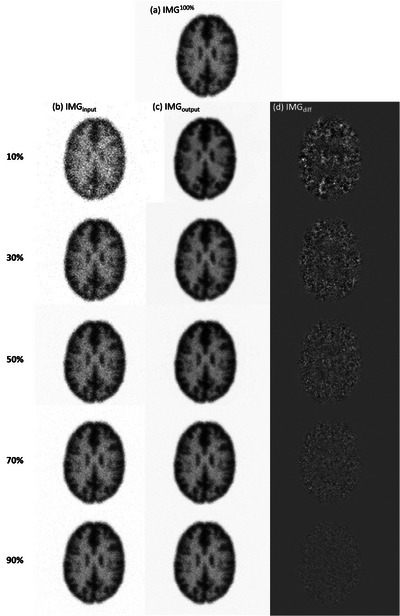
(a) IMG^100%^ for the Hoffman brain phantom, (b) IMG_input_ containing 10%, 30%, 50%, 70%, and 90% of the total photon counts (from top to bottom) and the corresponding (c) IMG_output_ and (d) IMG_diff_.

**FIGURE 7:**
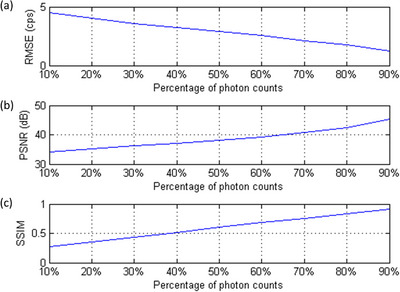
(a) RMSE, (b) PSNR, (c) SSIM between IMG^100%^ and IMG_output_ generated by denoising IMG_input_, which contained 10% to 90% of the total photon counts for the Hoffman brain phantom.

In Caffe, one training iteration means that one batch has been processed. According to our results, no obvious improvement in RMSE or PSNR was found after 80 000 iterations, so the CNN model trained for 100 000 iterations was used in this work. Figure 4a shows IMG^100%^ for the Shepp Logan phantom, while Figure 4b shows IMG_input_ containing 10%, 30%, 50%, 70%, and 90% of the total photon counts. The corresponding IMG_output_ and the difference image (IMG_diff_) between IMG^100%^ and  IMG_output_ are shown in Figure 4c and 4d, respectively. Figure 5 presents RMSE, PSNR, and SSIM between IMG^100%^ and IMG_output_ generated by denoising IMG_input_ that contained 10–90% of the total photon counts for the Shepp Logan phantom. Figure 6a shows IMG^100%^ for the Hoffman brain phantom, while Figure 6b shows IMG_input_ containing 10%, 30%, 50%, 70%, and 90% of the total photon counts. The corresponding IMG_output_ and IMG_diff_ are shown in Figure 6c and 6d, respectively. Figure 7 presents RMSE, PSNR, and SSIM between IMG^100%^ and IMG_output_ generated by denoising IMG_input_ that contained 10–90% of the total photon counts for the Hoffman brain phantom. Overall, the proposed CNN‐based denoising method could reduce the image noise in ^177^Lu planar scintigraphy, but IMG_output_ resulted from denoising IMG_input_ that contained fewer photon counts was less similar to IMG^100%^. The RMSE, PSNR, and SSIM for the Shepp Logan phantom were similar to those for the Hoffman brain phantom. This finding implied that anatomical complexity did not impact the imaging performance of IMG_output_ seriously.

### 3.2 CNN testing

**FIGURE 8:**
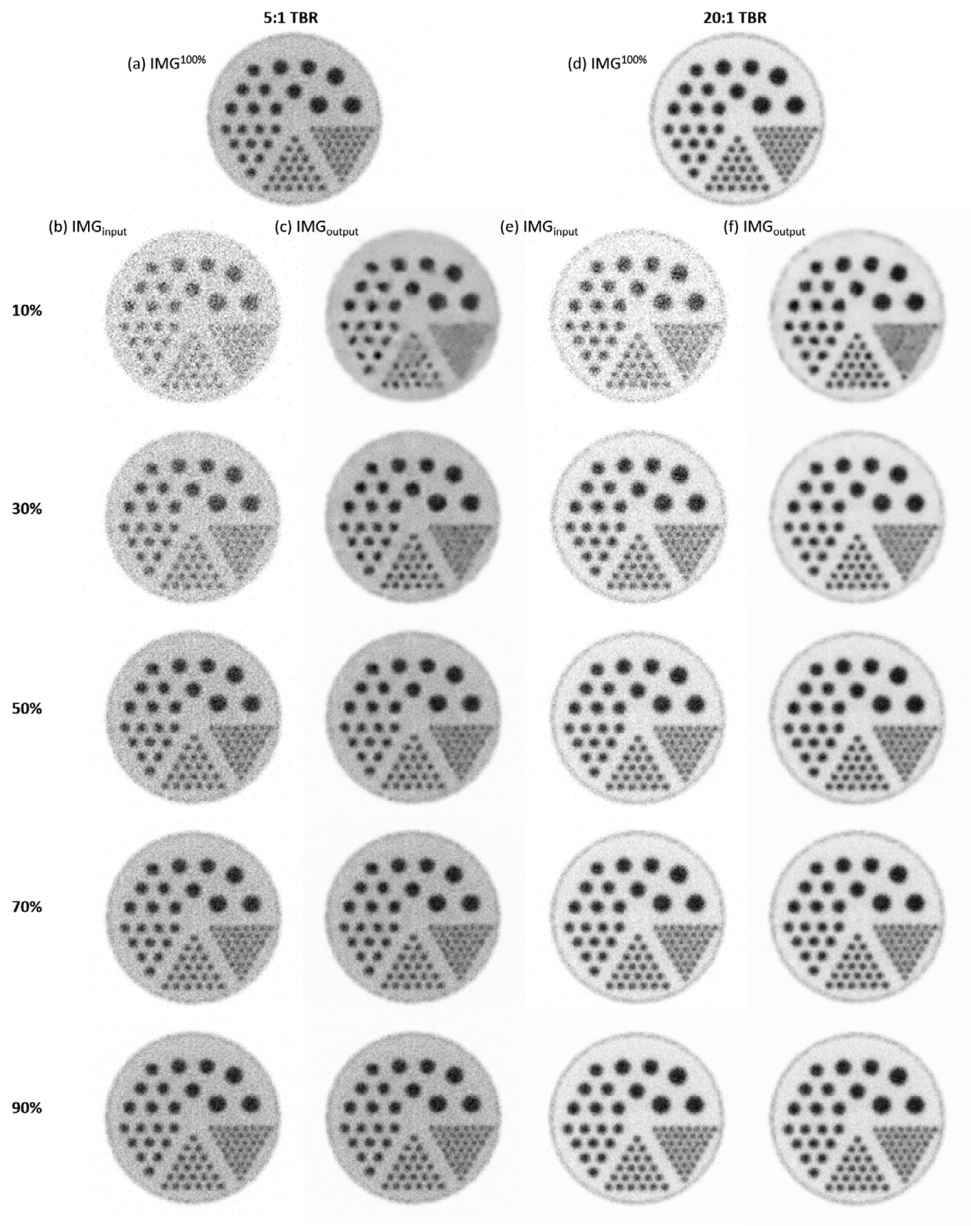
(a) IMG^100%^ for the Derenzo phantom with 5:1 TBR, (b) IMG_input_ containing 10%, 30%, 50%, 70%, and 90% of the total photon counts (from top to bottom), and (c) the corresponding IMG_output_. (d) IMG^100%^ for the Derenzo phantom with 20:1 TBR, (e) IMG_input_ containing 10%, 30%, 50%, 70%, and 90% of the total photon counts and (f) the corresponding IMG_output_.

Figure 8a shows IMG^100%^ for the Derenzo phantom with 5:1 TBR, while Figure 8b shows IMG_input_ containing 10%, 30%, 50%, 70%, and 90% of the total photon counts. The corresponding IMG_output_ are shown in Figure 8c. Figure 8d‐f shows IMG^100%^, IMG_input,_ and IMG_output_ for the Derenzo phantom with 20:1 TBR. The mean and SD of photon counts for the hot rods in Derenzo phantom with 5:1 TBR are summarized in Table 1. ${\mathrm{IMG}}_{\mathrm{input}}^{x\%}$ represented IMG_input_ that contained x% of the total photon counts, while ${\mathrm{IMG}}_{\mathrm{output}}^{x\%}$ represented IMG_output_ generated by denoising ${\mathrm{IMG}}_{\mathrm{input}}^{x\%}$. Moreover, Table 1 also shows the *P* value obtained by comparing the photon counts of the hot rods in Derenzo phantom with 5:1 TBR between IMG^100%^ and the others using two‐sample *t*‐test. Table 2 summarizes the mean and SD of photon counts for the Derenzo phantom with 20:1 TBR and the *P* value obtained from two‐sample *t*‐test. Based on naked eye observation, the 10 mm diameter rods in Derenzo phantom with 5:1 TBR can be discriminated from one another in ${\mathrm{IMG}}_{\mathrm{output}}^{50\%}$, ${\mathrm{IMG}}_{\mathrm{output}}^{70\%},$ and ${\mathrm{IMG}}_{\mathrm{output}}^{90\%}$, but not in ${\mathrm{IMG}}_{\mathrm{output}}^{10\%}$ and ${\mathrm{IMG}}_{\mathrm{output}}^{30\%}$. The 13 mm diameter rods in Derenzo phantom with 5:1 TBR can be discriminated from one another in ${\mathrm{IMG}}_{\mathrm{output}}^{30\%}$, ${\mathrm{IMG}}_{\mathrm{output}}^{50\%}$, ${\mathrm{IMG}}_{\mathrm{output}}^{70\%},$ and ${\mathrm{IMG}}_{\mathrm{output}}^{90\%}$, but not in ${\mathrm{IMG}}_{\mathrm{output}}^{10\%}$. In Table 1, statistically significant differences were found in ${\mathrm{IMG}}_{\mathrm{output}}^{10\%}$ ${\mathrm{IMG}}_{\mathrm{output}}^{20\%}$, ${\mathrm{IMG}}_{\mathrm{output}}^{30\%}$, ${\mathrm{IMG}}_{\mathrm{output}}^{40\%}$ for 10 mm diameter rods, and in ${\mathrm{IMG}}_{\mathrm{output}}^{10\%}$ for 13 mm diameter rods. With regard to the Derenzo phantom with 20:1 TBR, 10 mm diameter rods can be discriminated from one another in ${\mathrm{IMG}}_{\mathrm{output}}^{30\%}$, ${\mathrm{IMG}}_{\mathrm{output}}^{50\%}$, ${\mathrm{IMG}}_{\mathrm{output}}^{70\%},$ and ${\mathrm{IMG}}_{\mathrm{output}}^{90\%}$, but not in ${\mathrm{IMG}}_{\mathrm{output}}^{10\%}$. The 13 mm diameter rods in Derenzo phantom with 20:1 TBR can be discriminated from one another even in ${\mathrm{IMG}}_{\mathrm{output}}^{10\%}$. In Table 2, statistically significant differences were found in ${\mathrm{IMG}}_{\mathrm{output}}^{10\%}$, ${\mathrm{IMG}}_{\mathrm{output}}^{20\%}$, ${\mathrm{IMG}}_{\mathrm{output}}^{30\%}$, ${\mathrm{IMG}}_{\mathrm{output}}^{40\%}$, ${\mathrm{IMG}}_{\mathrm{output}}^{50\%}$, ${\mathrm{IMG}}_{\mathrm{output}}^{60\%}$ for 10 mm diameter rods, while no difference was found in any IMG_output_ for 13 mm diameter rods.

**TABLE 1:** Analysis results of photon counts for the hot rods in the Derenzo phantom with 5:1 TBR.

	10 mm			13 mm			17 mm			22 mm			28 mm			33 mm		
	Mean	SD	*P*	Mean	SD	*P*	Mean	SD	*P*	Mean	SD	*P*	Mean	SD	*P*	Mean	SD	*P*
IMG^100%^	57.96	10.27		64.40	13.24		78.60	14.32		85.99	15.38		93.69	14.26		94.27	15.40	
${\mathrm{IMG}}_{\mathrm{input}}^{10\%}$	5.83	2.53	*	6.37	2.77	*	7.71	2.92	*	8.65	3.30	*	9.57	3.23	*	9.44	3.09	*
${\mathrm{IMG}}_{\mathrm{input}}^{20\%}$	11.63	3.55	*	12.82	4.12	*	15.77	4.29	*	17.57	5.19	*	18.74	5.14	*	18.66	4.95	*
${\mathrm{IMG}}_{\mathrm{input}}^{30\%}$	17.42	4.61	*	19.17	5.31	*	23.61	5.71	*	26.32	6.62	*	28.14	6.31	*	28.14	6.20	*
${\mathrm{IMG}}_{\mathrm{input}}^{40\%}$	23.35	5.47	*	25.45	6.29	*	31.49	7.10	*	35.06	8.29	*	37.51	7.53	*	37.27	7.84	*
${\mathrm{IMG}}_{\mathrm{input}}^{50\%}$	29.24	6.30	*	31.68	7.36	*	39.07	8.31	*	43.65	9.33	*	46.65	8.20	*	46.87	9.09	*
${\mathrm{IMG}}_{\mathrm{input}}^{60\%}$	34.93	7.07	*	38.15	8.45	*	46.97	9.65	*	52.32	10.48	*	56.16	9.47	*	56.52	10.33	*
${\mathrm{IMG}}_{\mathrm{input}}^{70\%}$	40.58	7.96	*	44.82	9.57	*	55.15	10.96	*	60.81	11.60	*	65.43	10.84	*	65.98	11.59	*
${\mathrm{IMG}}_{\mathrm{input}}^{80\%}$	46.40	8.75	*	51.43	10.74	*	62.98	12.05	*	69.27	12.72	*	75.02	12.01	*	75.34	12.83	*
${\mathrm{IMG}}_{\mathrm{input}}^{90\%}$	52.17	9.48	*	57.98	11.99	*	70.73	13.16	*	77.67	13.90	*	84.39	13.39	*	84.64	14.14	*
${\mathrm{IMG}}_{\mathrm{output}}^{10\%}$	53.14	4.78	*	58.86	12.48	*	77.61	19.02	0.47	86.66	18.22	0.62	93.44	15.58	0.87	93.98	14.09	0.80
${\mathrm{IMG}}_{\mathrm{output}}^{20\%}$	54.37	6.13	*	62.19	12.06	0.01	77.28	14.66	0.27	87.16	18.57	0.40	92.16	14.30	0.29	94.01	14.80	0.83
${\mathrm{IMG}}_{\mathrm{output}}^{30\%}$	55.32	7.53	*	63.59	12.66	0.37	78.78	14.31	0.88	87.67	17.16	0.20	93.23	13.80	0.75	94.05	14.86	0.85
${\mathrm{IMG}}_{\mathrm{output}}^{40\%}$	55.85	7.85	*	62.64	11.84	0.04	77.81	14.31	0.50	87.14	16.51	0.37	93.29	13.29	0.77	92.80	14.49	0.21
${\mathrm{IMG}}_{\mathrm{output}}^{50\%}$	56.47	8.30	0.01	62.39	11.73	0.02	77.28	13.80	0.26	86.49	15.75	0.69	92.86	12.79	0.54	93.48	14.70	0.51
${\mathrm{IMG}}_{\mathrm{output}}^{60\%}$	56.74	8.72	0.03	62.50	11.75	0.03	77.65	14.28	0.42	86.77	15.67	0.53	93.51	12.90	0.90	94.25	14.56	0.99
${\mathrm{IMG}}_{\mathrm{output}}^{70\%}$	56.98	9.17	0.10	63.55	12.17	0.34	78.09	14.27	0.66	86.76	15.37	0.54	93.42	13.52	0.85	94.19	14.72	0.94
${\mathrm{IMG}}_{\mathrm{output}}^{80\%}$	57.39	9.59	0.34	63.87	12.57	0.55	78.48	14.14	0.91	86.37	15.18	0.76	93.80	13.81	0.94	94.08	14.85	0.87
${\mathrm{IMG}}_{\mathrm{output}}^{90\%}$	57.73	9.86	0.70	64.31	12.85	0.92	78.41	14.14	0.87	86.15	14.98	0.90	93.70	14.21	1.00	93.97	15.18	0.80

* The asterisk indicates a *P* value < 0.01.

**TABLE 2:** Analysis results of photon counts for the hot rods in the Derenzo phantom with 20:1 TBR.

	10 mm			13 mm			17 mm			22 mm			28 mm			33 mm		
	Mean	SD	*P*	Mean	SD	*P*	Mean	SD	*P*	Mean	SD	*P*	Mean	SD	*P*	Mean	SD	*P*
IMG^100%^	74.95	11.32		84.52	16.74		106.72	19.12		116.19	20.45		129.28	17.13		130.30	19.46	
${\mathrm{IMG}}_{\mathrm{input}}^{10\%}$	7.65	2.63	*	8.51	3.25	*	10.63	3.60	*	11.64	3.79	*	13.01	4.04	*	13.05	3.87	*
${\mathrm{IMG}}_{\mathrm{input}}^{20\%}$	15.26	4.24	*	16.81	5.08	*	21.28	5.45	*	23.27	6.03	*	26.01	5.69	*	26.08	6.12	*
${\mathrm{IMG}}_{\mathrm{input}}^{30\%}$	22.77	5.31	*	25.41	6.69	*	31.74	7.39	*	34.75	8.06	*	39.04	7.43	*	38.82	8.00	*
${\mathrm{IMG}}_{\mathrm{input}}^{40\%}$	30.11	6.08	*	33.72	8.24	*	42.66	8.95	*	46.27	9.76	*	51.84	8.90	*	52.12	9.60	*
${\mathrm{IMG}}_{\mathrm{input}}^{50\%}$	37.64	7.03	*	42.43	9.67	*	53.23	10.30	*	57.92	11.32	*	64.75	10.00	*	65.12	11.29	*
${\mathrm{IMG}}_{\mathrm{input}}^{60\%}$	45.19	7.89	*	50.94	11.18	*	64.27	12.18	*	69.38	13.35	*	77.32	11.48	*	78.32	12.92	*
${\mathrm{IMG}}_{\mathrm{input}}^{70\%}$	52.77	8.65	*	59.57	12.62	*	74.85	13.92	*	81.09	15.03	*	90.62	13.06	*	90.98	14.21	*
${\mathrm{IMG}}_{\mathrm{input}}^{80\%}$	60.24	9.44	*	67.89	14.11	*	85.23	15.85	*	92.84	16.95	*	103.28	14.65	*	104.32	15.85	*
${\mathrm{IMG}}_{\mathrm{input}}^{90\%}$	67.66	10.34	*	76.26	15.34	*	96.00	17.33	*	104.34	18.51	*	116.24	15.95	*	117.19	17.61	*
${\mathrm{IMG}}_{\mathrm{output}}^{10\%}$	66.56	8.87	*	83.48	20.41	0.42	104.45	23.34	0.20	114.11	25.62	0.27	127.60	20.36	0.37	128.09	22.72	0.19
${\mathrm{IMG}}_{\mathrm{output}}^{20\%}$	68.78	9.68	*	83.37	19.66	0.36	105.71	22.11	0.55	114.96	24.08	0.50	127.17	18.84	0.24	129.04	21.21	0.44
${\mathrm{IMG}}_{\mathrm{output}}^{30\%}$	70.82	9.89	*	84.33	20.09	0.88	105.01	21.38	0.31	115.62	23.99	0.75	129.85	19.23	0.75	129.45	21.49	0.60
${\mathrm{IMG}}_{\mathrm{output}}^{40\%}$	71.00	9.87	*	82.97	18.28	0.20	106.03	20.66	0.67	114.89	21.62	0.45	128.46	19.33	0.66	129.63	20.00	0.67
${\mathrm{IMG}}_{\mathrm{output}}^{50\%}$	72.25	10.75	*	83.89	18.14	0.61	104.87	19.01	0.24	114.93	20.97	0.45	128.61	18.00	0.70	129.57	19.87	0.64
${\mathrm{IMG}}_{\mathrm{output}}^{60\%}$	72.44	10.30	*	83.41	17.49	0.35	106.43	19.97	0.85	115.40	20.59	0.64	128.40	18.04	0.62	129.86	19.70	0.78
${\mathrm{IMG}}_{\mathrm{output}}^{70\%}$	73.13	10.68	0.01	84.20	17.51	0.79	106.45	19.62	0.87	115.43	20.71	0.65	129.40	17.79	0.95	129.47	19.39	0.59
${\mathrm{IMG}}_{\mathrm{output}}^{80\%}$	74.02	10.71	0.16	84.41	17.26	0.93	106.26	19.17	0.77	115.73	20.33	0.78	128.85	17.62	0.81	130.09	19.06	0.89
${\mathrm{IMG}}_{\mathrm{output}}^{90\%}$	74.58	11.01	0.58	84.60	16.89	0.94	106.42	19.05	0.85	115.67	20.22	0.75	128.89	17.38	0.82	130.01	19.18	0.85

* The asterisk indicates a *P* value < 0.01.

**FIGURE 9:**
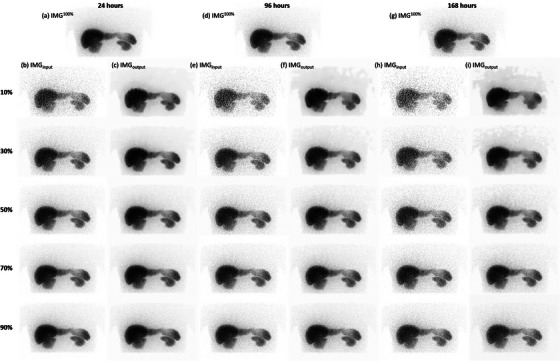
(a) IMG^100%^ for the NCAT phantom with activity distribution measured 24 h after injection, (b) IMG_input_ containing 10%, 30%, 50%, 70%, and 90% of the total photon counts (from top to bottom), and (c) the corresponding IMG_output_. (d) IMG^100%^ for the NCAT phantom with activity distribution measured 96 h after injection, (e) IMG_input_ containing 10%, 30%, 50%, 70%, and 90% of the total photon counts and (f) the corresponding IMG_output_. (g) IMG^100%^ for the NCAT phantom with activity distribution measured 168 h after injection, (h) IMG_input_ containing 10%, 30%, 50%, 70%, and 90% of the total photon counts and (i) the corresponding IMG_output_.

Figure 9a shows IMG^100%^ for the NCAT phantom with activity distribution measured 24 h after injection, while Figure 9b shows IMG_input_ containing 10%, 30%, 50%, 70%, and 90% of the total photon counts. The corresponding IMG_output_ are shown in Figure 9c. Figure 9d‐f show IMG^100%^, IMG_input_, and IMG_output_ for the NCAT phantom with activity distribution measured 96 h after injection. Figure 9g‐i show IMG^100%^, IMG_input_, and IMG_output_ for the NCAT phantom with activity distribution measured 168 h after injection. The mean and SD of photon counts for the source organs in NCAT phantom with activity distribution measured 24 h after injection were summarized in Table 3. Moreover, Table 3 also shows the *P* value obtained by comparing the photon counts of the source organs in NCAT phantom with activity distribution measured 24 h after injection between IMG^100%^ and the others using two‐sample *t*‐test. Analysis results for the NCAT phantom with activity distribution measured 96 h after injection are presented in Table 4, while those for the NCAT phantom with activity distribution measured 168 h after injection are presented in Table 5. Based on naked eye observation, changes in noise texture can be observed in ${\mathrm{IMG}}_{\mathrm{output}}^{10\%}$. Moreover, Table 5 shows there were statistically significant differences in ${\mathrm{IMG}}_{\mathrm{output}}^{10\%}$ for both right and left kidneys. On the other hand, no statistically significant differences were found in IMG_output_ for any other source organs in the NCAT phantom. However, a *P* value less than 0.05 was observed in spleen (24 h after injection) and left kidney (24, 96, and 168 h after injection) when comparing IMG^100%^ and ${\mathrm{IMG}}_{\mathrm{output}}^{20\%}$. Contrarily, all the *P* values were larger than 0.05 when comparing IMG^100%^ and ${\mathrm{IMG}}_{\mathrm{output}}^{30\%}$, indicating that the image denoising procedure should have little impact on the time activity curve derived from ^177^Lu planar scintigraphies that contained 30% of the total photon counts.

**TABLE 3:** Analysis results of photon counts for the source organs in the NCAT phantom with activity distribution measured 24 h after injection.

	Liver			Spleen			Right kidney			Left kidney		
	Mean	SD	*P*	Mean	SD	*P*	Mean	SD	*P*	Mean	SD	*P*
IMG^100%^	117.34	18.31		79.05	11.83		71.95	14.66		65.43	11.21	
${\mathrm{IMG}}_{\mathrm{input}}^{10\%}$	11.84	3.69	*	7.85	2.88	*	7.01	2.95	*	6.76	2.56	*
${\mathrm{IMG}}_{\mathrm{input}}^{20\%}$	23.56	5.62	*	15.45	4.28	*	14.33	4.52	*	13.60	3.86	*
${\mathrm{IMG}}_{\mathrm{input}}^{30\%}$	35.18	7.30	*	23.49	4.99	*	21.60	5.78	*	20.12	5.08	*
${\mathrm{IMG}}_{\mathrm{input}}^{40\%}$	46.95	9.15	*	31.38	6.19	*	28.77	7.07	*	26.43	6.07	*
${\mathrm{IMG}}_{\mathrm{input}}^{50\%}$	58.59	10.82	*	39.21	7.05	*	36.04	8.61	*	32.82	6.76	*
${\mathrm{IMG}}_{\mathrm{input}}^{60\%}$	70.29	12.24	*	46.87	8.05	*	43.08	9.76	*	39.30	7.76	*
${\mathrm{IMG}}_{\mathrm{input}}^{70\%}$	82.03	13.84	*	54.93	9.21	*	50.39	11.20	*	45.83	8.71	*
${\mathrm{IMG}}_{\mathrm{input}}^{80\%}$	93.93	15.34	*	62.91	10.33	*	57.62	12.49	*	52.37	9.41	*
${\mathrm{IMG}}_{\mathrm{input}}^{90\%}$	105.64	16.90	*	70.85	10.96	*	64.69	13.73	*	59.03	10.31	*
${\mathrm{IMG}}_{\mathrm{output}}^{10\%}$	118.75	16.19	0.04	76.24	8.41	0.01	70.30	14.06	0.16	66.48	8.37	0.17
${\mathrm{IMG}}_{\mathrm{output}}^{20\%}$	117.39	15.73	0.94	76.65	9.48	0.02	71.30	13.65	0.57	67.04	7.86	0.03
${\mathrm{IMG}}_{\mathrm{output}}^{30\%}$	117.09	16.48	0.71	77.37	9.21	0.11	71.45	13.67	0.66	66.66	9.02	0.12
${\mathrm{IMG}}_{\mathrm{output}}^{40\%}$	117.37	16.73	0.97	78.16	9.10	0.39	71.56	13.13	0.73	65.75	9.85	0.70
${\mathrm{IMG}}_{\mathrm{output}}^{50\%}$	117.10	17.15	0.73	78.35	9.85	0.51	72.02	13.94	0.95	65.28	9.67	0.85
${\mathrm{IMG}}_{\mathrm{output}}^{60\%}$	117.11	17.29	0.74	78.01	10.18	0.34	71.63	13.96	0.79	65.26	10.09	0.84
${\mathrm{IMG}}_{\mathrm{output}}^{70\%}$	117.21	17.63	0.85	78.32	10.85	0.51	71.97	14.47	0.99	65.03	10.52	0.64
${\mathrm{IMG}}_{\mathrm{output}}^{80\%}$	117.31	17.77	0.96	78.64	11.31	0.72	72.02	14.58	0.95	65.35	10.44	0.92
${\mathrm{IMG}}_{\mathrm{output}}^{90\%}$	117.34	18.12	1.00	78.73	11.48	0.78	71.89	14.79	0.96	65.54	10.82	0.90

* The asterisk indicates a *P* value < 0.01.

**TABLE 4:** Analysis results of photon counts for the source organs in the NCAT phantom with activity distribution measured 96 h after injection.

	Liver			Spleen			Right kidney			Left kidney		
	Mean	SD	*P*	Mean	SD	*P*	Mean	SD	*P*	Mean	SD	*P*
IMG^100%^	70.29	12.24		46.87	8.05		43.08	9.76		39.30	7.76	
${\mathrm{IMG}}_{\mathrm{input}}^{10\%}$	7.10	2.66	*	4.71	2.30	*	4.14	2.17	*	3.96	1.98	*
${\mathrm{IMG}}_{\mathrm{input}}^{20\%}$	14.16	4.07	*	9.48	3.18	*	8.45	3.32	*	8.12	2.77	*
${\mathrm{IMG}}_{\mathrm{input}}^{30\%}$	21.25	5.25	*	13.95	3.80	*	12.93	4.19	*	12.29	3.59	*
${\mathrm{IMG}}_{\mathrm{input}}^{40\%}$	28.19	6.33	*	18.64	4.46	*	17.32	5.04	*	16.23	4.32	*
${\mathrm{IMG}}_{\mathrm{input}}^{50\%}$	35.18	7.30	*	23.49	4.99	*	21.60	5.78	*	20.12	5.08	*
${\mathrm{IMG}}_{\mathrm{input}}^{60\%}$	42.24	8.38	*	28.17	5.84	*	25.80	6.56	*	23.97	5.73	*
${\mathrm{IMG}}_{\mathrm{input}}^{70\%}$	49.30	9.48	*	32.90	6.37	*	30.23	7.41	*	27.73	6.17	*
${\mathrm{IMG}}_{\mathrm{input}}^{80\%}$	56.30	10.58	*	37.60	6.83	*	34.71	8.33	*	31.55	6.60	*
${\mathrm{IMG}}_{\mathrm{input}}^{90\%}$	63.34	11.39	*	42.26	7.47	*	39.00	9.05	*	35.33	7.23	*
${\mathrm{IMG}}_{\mathrm{output}}^{10\%}$	70.49	8.72	0.63	45.84	4.91	0.12	41.43	8.26	0.02	38.04	4.91	0.69
${\mathrm{IMG}}_{\mathrm{output}}^{20\%}$	70.64	9.01	0.41	46.69	6.14	0.80	42.12	8.44	0.19	39.50	4.78	0.04
${\mathrm{IMG}}_{\mathrm{output}}^{30\%}$	70.70	10.35	0.36	46.07	5.85	0.25	42.71	8.49	0.61	40.39	5.61	0.33
${\mathrm{IMG}}_{\mathrm{output}}^{40\%}$	70.43	10.42	0.76	46.14	5.93	0.30	43.01	8.66	0.92	39.84	6.41	0.30
${\mathrm{IMG}}_{\mathrm{output}}^{50\%}$	70.25	10.75	0.92	46.68	6.36	0.79	43.06	8.81	0.98	39.89	6.69	0.53
${\mathrm{IMG}}_{\mathrm{output}}^{60\%}$	70.39	11.07	0.84	46.87	7.04	0.99	42.91	9.02	0.82	39.66	6.96	0.93
${\mathrm{IMG}}_{\mathrm{output}}^{70\%}$	70.33	11.76	0.93	46.91	7.44	0.96	43.06	9.33	0.97	39.24	7.26	0.94
${\mathrm{IMG}}_{\mathrm{output}}^{80\%}$	70.33	12.02	0.94	46.95	7.33	0.91	43.35	9.60	0.74	39.34	7.15	0.89
${\mathrm{IMG}}_{\mathrm{output}}^{90\%}$	70.34	12.09	0.91	46.97	7.73	0.90	43.32	9.63	0.76	39.21	7.54	0.69

* The asterisk indicates a *P* value < 0.01.

**TABLE 5:** Analysis results of photon counts for the source organs in the NCAT phantom with activity distribution measured 168 h after injection.

	Liver			Spleen			Right kidney			Left kidney		
	Mean	SD	*P*	Mean	SD	*P*	Mean	SD	*P*	Mean	SD	*P*
IMG^100%^	46.95	9.15		31.38	6.19		28.77	7.07		26.43	6.07	
${\mathrm{IMG}}_{\mathrm{input}}^{10\%}$	4.78	2.17	*	3.14	1.89	*	2.72	1.82	*	2.63	1.65	*
${\mathrm{IMG}}_{\mathrm{input}}^{20\%}$	9.43	3.23	*	6.31	2.54	*	5.59	2.59	*	5.32	2.32	*
${\mathrm{IMG}}_{\mathrm{input}}^{30\%}$	14.16	4.07	*	9.48	3.18	*	8.45	3.32	*	8.12	2.77	*
${\mathrm{IMG}}_{\mathrm{input}}^{40\%}$	18.96	4.86	*	12.48	3.67	*	11.39	3.95	*	10.92	3.35	*
${\mathrm{IMG}}_{\mathrm{input}}^{50\%}$	23.56	5.62	*	15.45	4.28	*	14.33	4.52	*	13.60	3.86	*
${\mathrm{IMG}}_{\mathrm{input}}^{60\%}$	28.19	6.33	*	18.64	4.46	*	17.32	5.04	*	16.23	4.32	*
${\mathrm{IMG}}_{\mathrm{input}}^{70\%}$	32.85	7.02	*	21.89	4.82	*	20.21	5.53	*	18.84	4.87	*
${\mathrm{IMG}}_{\mathrm{input}}^{80\%}$	37.55	7.71	*	25.07	5.41	*	23.04	5.98	*	21.41	5.22	*
${\mathrm{IMG}}_{\mathrm{input}}^{90\%}$	42.24	8.38	*	28.17	5.84	*	25.80	6.56	*	23.97	5.73	*
${\mathrm{IMG}}_{\mathrm{output}}^{10\%}$	47.42	5.96	0.12	30.16	4.16	0.02	26.86	5.91	*	24.49	3.44	*
${\mathrm{IMG}}_{\mathrm{output}}^{20\%}$	46.89	6.47	0.86	30.44	4.11	0.07	27.99	5.79	0.13	25.54	3.69	0.02
${\mathrm{IMG}}_{\mathrm{output}}^{30\%}$	46.99	6.75	0.89	30.72	4.67	0.22	27.83	6.19	0.08	25.96	3.99	0.24
${\mathrm{IMG}}_{\mathrm{output}}^{40\%}$	47.37	7.25	0.20	30.78	4.62	0.27	28.33	6.22	0.42	26.67	4.36	0.57
${\mathrm{IMG}}_{\mathrm{output}}^{50\%}$	47.04	7.70	0.79	30.77	5.24	0.29	28.52	6.49	0.65	26.85	4.74	0.32
${\mathrm{IMG}}_{\mathrm{output}}^{60\%}$	46.93	7.98	0.95	31.02	5.25	0.53	28.83	6.47	0.92	26.81	5.10	0.39
${\mathrm{IMG}}_{\mathrm{output}}^{70\%}$	46.92	8.31	0.93	31.12	5.32	0.65	28.72	6.62	0.93	26.51	5.50	0.86
${\mathrm{IMG}}_{\mathrm{output}}^{80\%}$	46.92	8.59	0.93	31.25	5.72	0.82	28.78	6.75	0.99	26.67	5.59	0.60
${\mathrm{IMG}}_{\mathrm{output}}^{90\%}$	46.92	8.79	0.94	31.30	6.02	0.89	28.66	6.93	0.84	26.61	5.92	0.70

* The asterisk indicates a *P* value < 0.01.

## 4 DISCUSSION

The detection of gamma‐emitting radionuclide by planar imaging is a random process that is governed by Poisson statistics. Reducing scan time can increase patient comfort, but it also produces statistical noise in the resulting images and may thus lead to bias in the measurements of organ activity. Image denoising is a task that recovers a clean image from a noisy version. In nuclear medicine imaging, several physical factors were found to have influence on radioactivity quantification, including spatial resolution, partial volume effect and statistical noise.^21^ Hence, a successful denoising method for ^177^Lu planar scintigraphy should be able to suppress statistical noise without causing serious resolution loss. In general, denoising methods can be divided into two main categories: model‐based optimization methods and discriminative learning methods.^22^ The most actively studied discriminative methods are deep neural networks, in which differences can be observed among various network models. Minarik et al. used a denoising convolutional neural network (DnCNN), which has three convolution layers to reduce statistical noise in whole‐body bone scintigraphy and decrease scan time.^23^ According to their results, the DnCNN enables reducing the scanning time by half and still obtaining good accuracy for bone metastasis assessment. Olia et al. used a generative adversarial network (GAN) to reduce statistical noise in SPECT myocardial perfusion images, leading to a decrease in patient radiation dose.^24^ Their results demonstrated that GAN could recover the underlying information in 1/2‐dose and 1/4‐dose SPECT images. Because the architecture of GAN is more complex than that of DnCNN, it may explain why GAN achieves better denoising performance. On the other hand, it is rather challenging to train a GAN model due to vanishing gradient, mode collapse, and non‐convergence. The architecture of DenseNet used in this work is more complex than that of DnCNN. Moreover, the dense skip connections make network training easier and generalization performance better, so this study investigated the potential of using DenseNet to reduce scan time in ^177^Lu planar scintigraphy. The DenseNet model proposed by Tong et al. was originally designed for super‐resolution,^13^ which has been adopted by Kim et al. to enhance signal and noise performance in ^99m^Tc SPECT imaging.^25^ Their super‐resolution method produced better image quality compared to FBP and OS‐EM in terms of contrast‐to‐noise ratio (CNR), coefficient of variation (COV) and PSNR. Besides resolution recovery, DenseNet has been proven to be an effective type of neural network to reduce the image noise due to low‐dose CT scans.^26^ However, to the best of our knowledge, it has not been used for image denoising in ^177^Lu planar scintigraphy. Since both image detail and statistical noise are high‐frequency components, the process of denoising would inevitably lead to some loss of spatial resolution.^27^ Therefore, the DenseNet model with dense skip connections was used in this work to evaluate its efficacy in balancing the tradeoff between noise reduction and resolution degradation.

For CNN‐based denoising methods, the datasets for model training are crucial to the resulting performance.^23^, ^28^ Monte Carlo techniques are essential tools in nuclear medicine imaging and have been applied to develop image correction and processing techniques for over 50 years.^29^ Because simulation can prevent biases introduced into deep learning models due to insufficient training samples or imperfect data quality that are often encountered in real patient scans, simulated ^177^Lu planar scintigraphies were used for CNN training and testing in this work. The Shepp Logan phantom and the Hoffman brain phantom are both 3D phantoms but have very different anatomical complexity, so they were used for model training to ensure sufficient data diversity. With regards to model testing, the Derenzo phantom was used to investigate the impact of scan time reduction on the photon counts of hot rods with different sizes, while the NCAT phantom was used to investigate the impact of scan time reduction on the photon counts of source organs measured at different time points. Based on Derenzo evaluation, it was found that the imaging performance of IMG_output_ was degraded when increasing the background activity. Nevertheless, no statistically significant difference was found in photon counts between IMG^100%^ and ${\mathrm{IMG}}_{\mathrm{output}}^{30\%}$ for hot rods larger than 13 mm in diameter. Although statistically significant differences were found in ${\mathrm{IMG}}_{\mathrm{output}}^{30\%}$ for 10‐mm diameter rods, the difference was 4.55% for the Derenzo phantom with 5:1 TBR and 5.51% for the Derenzo phantom with 20:1 TBR. In ${\mathrm{IMG}}_{\mathrm{output}}^{50\%}$, the difference was reduced to 2.57% and 3.60% for the Derenzo phantom with 5:1 TBR and 20:1 TBR, respectively. According to MIRD schema, the activity in source organ *k* is given by the expression:

(5) $$A=\;F\sqrt{\frac{I_{A}I_{P}}{T}}\frac{f}{C}$$

*I_A_* and *I_P_* are the anterior image and the posterior image of the source region in planar scintigraphic imaging ($\frac{\mathrm{counts}}{\sec}$). T is the transmission factor, and *C* is the imaging system calibration factor ($\frac{\mathrm{counts}/\sec}{\mathrm{Bq}}$). *F* is the background correction factor, and *f* provides the correction for the source region attenuation and source thickness.^6^, ^7^, ^8^, ^9^ Hence, an accurate determination of the photon counts in Lu‐177 planar scintigraphy is crucial for calculating the source activity, which is required for calculating the absorbed dose in target organ *j* (see Equation 1). Based on NCAT phantom evaluation, the changes in noise texture observed in ${\mathrm{IMG}}_{\mathrm{output}}^{10\%}$ were not found in ${\mathrm{IMG}}_{\mathrm{output}}^{30\%}.$ Moreover, no significant difference was found in photon counts between IMG^100%^ and ${\mathrm{IMG}}_{\mathrm{output}}^{30\%}$ for source organs measured at all time points. Therefore, the proposed CNN‐based denoising method enables a reduction of up to 70% in scan time, or an increase in scan speed by three times. Increasing the scan speed to 30 cm/min in whole‐body planar scintigraphy shortens the procedure to approximately 7 min per planar scan. The proposed method has the potential to improve patient comfort while maintaining dosimetry accuracy for ^177^Lu‐based peptide receptor radionuclide therapy, which may facilitate personalized dosimetry in clinical practice. However, further patient studies are needed to confirm its clinical efficacy.

## 5 CONCLUSION

While reducing scan time in nuclear medicine imaging increases patient comfort, it also creates statistical noise in images and affects activity measurement accuracy. Therefore, this study investigated the potential of using DenseNet to reduce scan time in ^177^Lu planar scintigraphy. Our results indicate that the proposed CNN‐based denoising method can reduce scan time by up to 70% for objects larger than 13 mm. This could greatly facilitate personalized dosimetry in clinical practice for ^177^Lu‐based peptide receptor radionuclide therapy.

## AUTHOR CONTRIBUTIONS

Conceptualization, Ching‐Ching Yang, Kuan‐Yin Ko, and Pei‐Yao Lin; methodology, Ching‐Ching Yang, Kuan‐Yin Ko, and Pei‐Yao Lin; software, Ching‐Ching Yang; validation, Ching‐Ching Yang; formal analysis, Ching‐Ching Yang and Pei‐Yao Lin; investigation, Ching‐Ching Yang and Kuan‐Yin Ko; resources, Ching‐Ching Yang and Kuan‐Yin Ko; data curation, Ching‐Ching Yang, Kuan‐Yin Ko, and Pei‐Yao Lin; writing, Ching‐Ching Yang, visualization, Ching‐Ching Yang; supervision, Ching‐Ching Yang and Kuan‐Yin Ko; project administration, Ching‐Ching Yang and Kuan‐Yin Ko.

## CONFLICT OF INTEREST STATEMENT

The authors declare no conflicts of interest.
